# Multidisciplinary Prehabilitation and Postoperative Rehabilitation for Avoiding Complications in Patients Undergoing Resection of Colon Cancer: Rationale, Design, and Methodology of the ONCOFIT Study

**DOI:** 10.3390/nu14214647

**Published:** 2022-11-03

**Authors:** Francisco J. Amaro-Gahete, Javier Jurado, Andrea Cisneros, Pablo Corres, Andres Marmol-Perez, Francisco J. Osuna-Prieto, Manuel Fernández-Escabias, Estela Salcedo, Natalia Hermán-Sánchez, Manuel D. Gahete, Virginia A. Aparicio, Cristina González-Callejas, Benito Mirón Pozo, Jonatan R. Ruiz, Teresa Nestares, Almudena Carneiro-Barrera

**Affiliations:** 1 PROFITH (PROmoting FITness and Health through Physical Activity) Research Group, Sport and Health University Research Institute (iMUDS), Department of Physical Education and Sport, Faculty of Sport Sciences, University of Granada, 18007 Granada, Spain; 2 Department of Physiology, Faculty of Medicine, EFFECTS-262 Research Group, University of Granada, 18016 Granada, Spain; 3 Centro de Investigación Biomédica en Red Fisiopatología de la Obesidad y Nutrición (CIBERobn), Instituto de Salud Carlos III, 28029 Madrid, Spain; 4 Service of Surgery, Hospital Universitario Clínico San Cecilio, 18016 Granada, Spain; 5 Department of Physical Education and Sport, Faculty of Education and Sport-Physical Activity and Sport Sciences Section, University of the Basque Country (UPV/EHU), 01007 Vitoria-Gasteiz, Spain; 6 Department of Analytical Chemistry, University of Granada, 18071 Granada, Spain; 7 Service of Clinical Psychology, Hospital Universitario Clínico San Cecilio, 18016 Granada, Spain; 8 Maimónides Biomedical Research Institute of Córdoba, 14004 Córdoba, Spain; 9 Department of Cell Biology, Physiology and Immunology, University of Córdoba, 14004 Córdoba, Spain; 10 Reina Sofía University Hospital, 14004 Córdoba, Spain; 11 Department of Physiology, Faculty of Pharmacy, University of Granada, 18016 Granada, Spain; 12 Centro de Investigación Biomédica (CIBM), Instituto de Nutrición y Tecnología de los Alimentos “José Mataix” (INYTA), Universidad de Granada, 18016 Granada, Spain; 13 Instituto de Investigación Biosanitaria, ibs.Granada, 18016 Granada, Spain; 14 Department of Psychology, Universidad Loyola Andalucía, 41007 Seville, Spain

**Keywords:** tumor, exercise, nutrition, oncology, cardiovascular risk factors, public health

## Abstract

ONCOFIT is a randomized clinical trial with a two-arm parallel design aimed at determining the influence of a multidisciplinary Prehabilitation and Postoperative Program (PPP) on post-surgery complications in patients undergoing resection of colon cancer. This intervention will include supervised physical exercise, dietary behavior change, and psychological support comparing its influence to the standard care. Primary and secondary endpoints will be assessed at baseline, at preoperative conditions, at the end of the PPP intervention (after 12 weeks) and 1-year post-surgery, and will include: post-surgery complications (primary endpoint); prolonged hospital length of stay; readmissions and emergency department call within 1-year after surgery; functional capacity; patient reported outcome measures targeted; anthropometry and body composition; clinical/tumor parameters; physical activity levels and sedentariness; dietary habits; other unhealthy habits; sleep quality; and fecal microbiota diversity and composition. Considering the feasibility of the present intervention in a real-life scenario, ONCOFIT will contribute to the standardization of a cost-effective strategy for preventing and improving health-related consequences in patients undergoing resection of colon cancer with an important clinical and economic impact, not only in the scientific community, but also in clinical practice.

## 1. Introduction

Colon cancer is currently diagnosed in ~1.1 million patients/year, accounting for more than 500,000 deaths per year and making this tumor the 5th most common type of cancer worldwide in 2020 [1]. Nowadays, surgical resection is considered the elective therapy, in that it represents a curative treatment for colon cancer [2]. However, surgery is a stressful procedure that may involve numerous hematological, immunological and endocrinological complications [3]. Given that colon cancer is averagely diagnosed at ~70 years [1], it is relatively common that these patients present several age-related disturbances and comorbidities, which increases the incidence of further postoperative complications and extends hospitalization stays [4]. Indeed, postoperative complications are registered in >50% of patients, increasing the incidence of morbi-mortality rates and health care economic burden, while decreasing the patient’s health-related quality of life [5].

Risk factors for such undesirable events are closely related to inappropriate lifestyle behaviors, including decreased physical activity and fitness levels, dietary unhealthy habits (e.g., increased consumption of ultra-processed food), alcohol ingestion, sleep disturbances, or tobacco [6], all of them related to subsequent increments of obesity rates [7] and the higher incidence of chronic cardiovascular diseases [8]. Recent studies have suggested that the implementation of multidisciplinary prehabilitation interventions-procedures aimed to enhance the functional capacity of individuals, enabling them to withstand an incoming stressor-to counteract age-related patients’ vulnerability before surgery (i.e., physical, dietary, and psychological programs) may be a potential strategy not only for improving human physiological reserve, but also to accelerate postoperative recovery [9,10,11]. However, there is no strong evidence supporting that these hypothetical functional benefits would be related to enhancements in postoperative morbidity and mortality [12].

Important entities, such as the World Health Organization [13] or the American Cancer Society [14], have developed clinical guidelines focused on cancer survivors for health maintenance, recommending: (i) to complete at least a total of 150 (moderate intensity) or 75 (vigorous intensity) minutes of aerobic physical activity combined with two sessions/week of strength training [13]; (ii) to follow a healthy dietary pattern based on the Mediterranean diet [15]; and (iii) to have phycological support [16] to improve both physical and mental health. These strategies are appropriate to restore functional capacity after surgery [17], and to reduce risk factors for cancer recurrence or the appearance of chronic cardiometabolic diseases [18]. Nevertheless, there is a lack of oncology rehabilitation programs after colon cancer surgery and, therefore, well-designed randomized clinical trials are needed to test their potential efficacy.

A recent study by Carli et al. [19] compared the effect of a 4-week prehabilitation program vs. 4-week postoperative rehabilitation intervention on postoperative complications in patients with colorectal cancer resection, concluding that there was no difference between programs on post-surgery outcomes. Nonetheless, to the best of our knowledge, the combined effect of a Prehabilitation in addition to Postoperative Programs (PPP) on post-surgery complications and the functional capacity of patients undergoing resection of colon cancer remains unclear, being also still unknown their optimal duration. Therefore, the overall objective of the ONCOFIT randomized clinical trial is to determine the effect of a multidisciplinary prehabilitation (i.e., 4 weeks before surgery) plus postoperative (i.e., 12 weeks after surgery) program on post-surgery complications (primary endpoint-assessed by the Comprehensive Complication Index (CCI)) in patients undergoing resection of colon cancer. This intervention will include supervised concurrent physical exercise (i.e., aerobic plus strength training) based on the current physical activity recommendations provided by the World Health Organization, dietary behavior change, and psychological support, comparing its influence to the usual care. We will additionally investigate the effects of the above-mentioned intervention on: prolonged hospital length of stay, readmissions, and emergency department call within 1-year after surgery; functional capacity (assessed by physical fitness); patient reported outcome measures targeted (i.e., health-related quality of life, depression symptoms, anxiety symptoms, optimistic/pessimistic status, affect symptoms and satisfaction symptoms); anthropometry and body composition; clinical/tumor parameters (i.e., blood parameters, clinical characterization, tumor biomarkers, circulating biomarkers, and blood pressure); physical activity levels and sedentariness; dietary habits; other unhealthy habits (e.g., tobacco and alcohol consumption); sleep quality; and fecal microbiota diversity and composition.

## 2. Materials and Methods

### 2.1. Study Design

**Figure 1:**
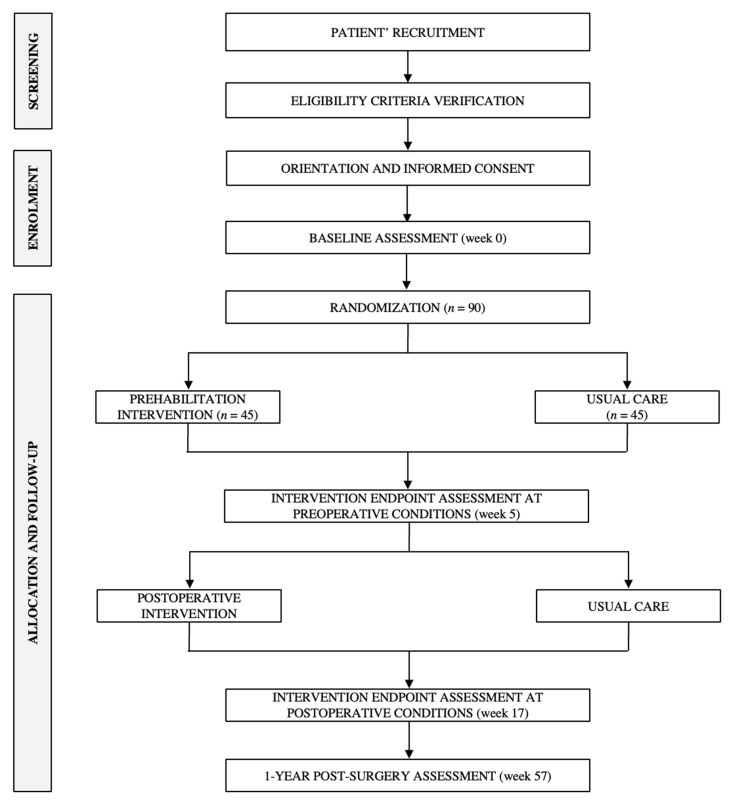
Flow diagram of the ONCOFIT study patients.

The ONCOFIT study (ClinicalTrials.gov ID: NCT05379205) is a randomized clinical trial with a two-arm parallel design. Patients will be randomly allocated to a usual care (i.e., control group) or a PPP (4 weeks + 12 weeks) group. The study was designed following the Ethical Principles for Medical Research Involving Human Subjects contained in the Declaration of Helsinki (last revised version, 2013) and has been approved by The Human Research Ethics Committee of the “Junta de Andalucía” (2019529142937). All patients will receive oral information on the study intervention and measurements and, subsequently, they will be instructed to voluntary sign a written informed consent before their enrolment. Individuals diagnosed with colon cancer that meet the inclusion criteria will be recruited from the “San Cecilio University Hospital” (Granada, Spain). Both baseline and follow-up data collection and the PPP intervention will be conducted in two settings: (i) the above-mentioned Hospital, and (ii) the Sport and Health University Research Institute (iMUDS, University of Granada, Granada, Spain). The patient flow diagram from the recruitment to the randomization stages can be found in Figure 1.

### 2.2. Participants and Selection Criteria

Eligible individuals will be adults diagnosed with colon cancer. A complete list of the study inclusion and exclusion criteria can be found in Table 1. A complete medical history and examination will be conducted for all potential participants, checking whether they meet the inclusion criteria and ensuring no harm-related conditions derived from both intervention and assessment protocols. We will contract a clinical trial liability insurance for the ONCOFIT study, action that provides (i) financial and legal protection to the research team, and (ii) economic compensation to the enrolled patients if any damage or injury occur.

### 2.3. Recruitment and Randomization

After concluding the baseline assessment, eligible patients will be randomly assigned (1:1 allocation) to either a usual care (control) group or a PPP group by means of computer generated simple, unrestricted randomization [20]. We will inform patients of which study arm they have been assigned, requesting them not to unveil their allocation to those responsible for further assessments. We will also address different strategies of blinding for the study participants and researchers, where feasible, in order to avoid potential bias related to the lack of patients’ blinding, treatment counsellors, or outcome evaluators affecting data validity. Research staff in charge of data collection and statistical analyses will be therefore blinded to the participants’ allocation assignments at follow-up, while patients will not receive information about the study manuals and hypothesis. Rigorous standardized procedures for data collection and intervention will be implemented when blinding will be not possible for avoiding bias and guaranteeing external and internal validity [21]. Both the investigator and the surgeon will verify patient eligibility. If surgery is the elective procedure, patients will be subsequently checked, ensuring that they meet the inclusion criteria of the present study. Patients will receive a phone call to arrange an appointment in which they (i) provide oral and written consent to participate in the trial after details clarification of the study rationale and aims, and (ii) schedule testing, intervention, and surgery days.

#### Evaluation of Integrity, Compliance of the Intervention and Patients’ Retention/Adherence

To appropriately guarantee the integrity and fidelity of the present intervention, we will adopt specific strategies regarding patient monitorization and assessment standardization to ensure external and internal validity of the clinical trial [22,23].

On the one hand, we will create a comprehensive hand-book for the ONCOFIT study’s research staff (i.e., medical doctors and surgeons, training specialists, nutritionists, and phycologists) indicating the appropriate details about testing days and intervention sessions (e.g., objectives, contents, activities, materials, or tasks). On the other hand, the fidelity of the intervention will be ensured by using previously developed manuals and protocols, and providing information to the patients in different formats.

Although all patients will be allowed to withdraw during the randomized controlled trial development, several strategies will be implemented to reduce drop-out rates and to keep the adherence to the proposed intervention. Firstly, an experienced multidisciplinary team composed of medical doctors and surgeons, training specialists, nutritionists, and phycologists, will be available to perform the intervention, circumstance that guarantee its successfully development. Exercise sessions will be performed in a cutting-edge and well-equipped research center (Laboratory of Exercise Physiology, Sport and Health University Research Institute (iMUDS), Granada, Spain), accompanied by music in reduced groups (i.e., less than three patients) to ensure the correct assistance in all cases. Dietary behavior and phycological support sessions will also be conducted in the same research center, using digital media in well-conditioned rooms. We will also prevent vacation periods during the intervention, and the research staff (independently of its precedence) will constantly give support to the patients in all intervention stages. Finally, we will systematically record patients’ attendance to each intervention session performing phone-calls to know absence causes and, subsequently, to schedule a new appointment to retrieve the lost session.

### 2.4. Intervention Description

The study rationale is based on previous research evidence supporting the notion that both prehabilitation [9,10,11,12] and postoperative programs [17,19] can independently improve general health and the quality of life in patients undergoing resection of colon cancer. Considering their recognized limitations, and with the final objective of reducing post-surgery complications and further health-related parameters, we will implement a PPP intervention (i.e., 4-week prehabilitation plus 12-week postoperative program) with three complementary modules: (i) physical exercise, (ii) dietary behavior change, and (iii) psychological support.

The Transtheoretical Model of Health Behavior Change proposed by Prochaska and Diclemente [24]—a well-known model of behavior change that aimed to promote sustainable health-related habits through different processes, stages and strategies—will be the basis of the present multidisciplinary intervention. Principles and processes such as stimulus control, decisional balance, self-reevaluation, goal-setting, consciousness raising, self-efficacy, contingency management, self-monitoring, and counterconditioning [24] will be implemented in the three modules of the PPP intervention.

#### 2.4.1. Physical Exercise Intervention

Recent studies have demonstrated the potential health-related benefits derived from physical exercise interventions in patients with colon cancer undergoing surgery before [9,10,11,12] and after the resection procedure [17,19]. However, these interventions are dissimilar in terms of (i) physical exercise modality (e.g., aerobic, strength or concurrent training), (ii) volume (e.g., low vs. high), (iii) intensity (e.g., moderate, vigorous, or high intensity), (iv) frequency (e.g., two or three sessions/week), and (v) training density and recovery. Moreover, no detailed information is available regarding the above-mentioned parameters or training periodization in most studies implementing this type of intervention, which limits replication strategies. Furthermore, no previous study has tested whether the implementation of a PPP program provides additional effects on post-surgery complications and further health-related parameters and the quality of life in patients undergoing resection of colon cancer, in addition to those obtained by a prehabilitation or a postoperative program alone. Therefore, specific features of our PPP intervention are provided below.

##### Volume

Since the transferability of the study results to the general population is mandatory for us, the volume of the present intervention will be based on the physical activity recommendations provided by the World Health Organization (i.e., >75 min/week at vigorous/high intensity of aerobic training combined with >2 session of strength training) independently of the intervention phase.

##### Intensity

Most of the previous physical exercise interventions in this field have been performed at moderate intensity, obtaining promising results [9,10,11,17,19]. Nevertheless, it has been suggested that implementing a high intensity interval training (HIIT) methodology may result in similar or even greater improvements, while considerably reducing training volume [12]. Indeed, HIIT has been positioned as an effective method to improve several health-related physiological parameters in both healthy individuals and patients [25,26,27,28]. Therefore, the present PPP intervention sessions will be based on HIIT in both aerobic and strength training parts.

Aerobic exercise will consist of 4 min intervals at >85% peak heat rate (pHR) interposed by 3 min of active recovery at 65–75% of pHR. To stablish strength training intensity, the maximum rating perceived exertion (i.e., RPE scale (0–10)) will be used prescribing an intensity of >8 RPE as the target, which could be modulated by the execution speed and band resistance. Heart rate will be additionally monitored in all training sessions.

##### Frequency

The training session frequency of the ONCOFIT intervention will be three times/week based on the recommendations of previous studies [25,26,27,28]. We will therefore establish a resting period of ≥48 h between sessions.

##### Type of Exercise

Aerobic HIIT training sessions will consist of uphill treadmill walking with personalized slopes. Strength HIIT training sessions will be organized in a circuit form and will be composed by a combined selection of upper and lower body exercises (i.e., basic movement patterns) using as the primary resistance elastic bands and the patient’s body weight (i.e., band horizontal/vertical pulls, core anti-extension, squat, vertical/push-ups press, monster walker, glutes bridge). We will propose three levels of exercises’ difficulty, based on resistance band and the increasing complexity of the basic movement pattern task.

##### Training Load Variation

Considering that the majority of the potential recruited patients will have a sedentary status, we propose a gradual progression of both training volume and intensity, aiming to control the physical exercise dose.

Patients will begin with a dose of 36 min/week at 80–85% pHR for aerobic HIIT (84 min/week of total aerobic training), while a dose of 60 min/week at 7–8 RPE will be implemented in strength HIIT until they progressively reach the preestablished volume and intensity.

##### Training Periodization

**Figure 2:**
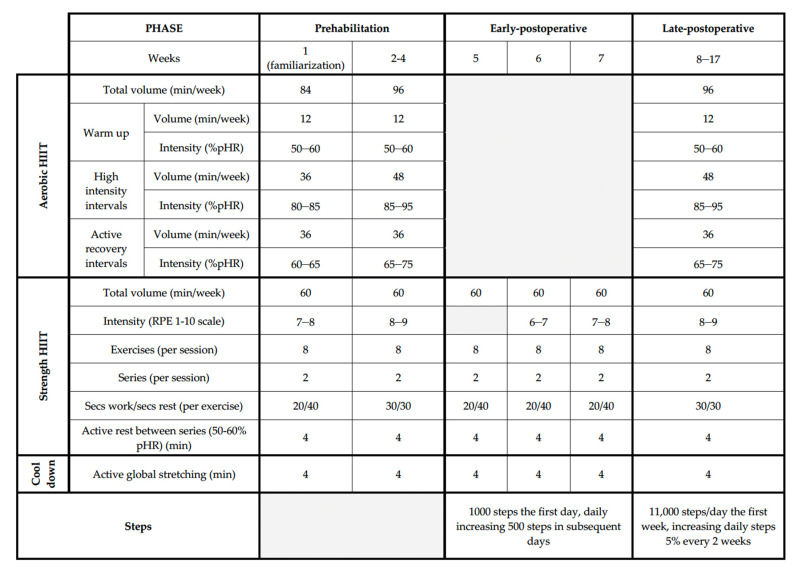
Training periodization of the ONCOFIT study. Abbreviations: HIIT; High Intensity Interval Training, pHR; peak Heart Rate, RPE; Rating Perceived Exertion.

Training periodization is divided into three subsequent phases: (i) prehabilitation phase (4 weeks), (ii) early-postoperative phase (3 weeks), and (iii) late-postoperative phase (9 weeks) (Figure 2).

##### Prehabilitation Phase

The first week of this phase is designed as a familiarization period in which the patients learn: (i) the structure and organization of the training sessions, and (ii) the main movement patterns (i.e., horizontal, and vertical pulls and push, squat, hinge, and core stability). An aerobic HIIT session total volume will be 28 min organized as follow: (i) warm-up for 4 min at 50–60% pHR, and (ii) three 4 min intervals at 80–85% of pHR interposed by 4 min of active recovery at 60–65% of pHR. Strength HIIT total volume will be 20 min organized as follow: two circuit trainings set (8 min × 2 = 16 min) of eight exercises (20 s work/40 s rest) at 7–8 RPE, with an active between-set rest of 4 min performed at 50–60% of pHR. A cool-down protocol (i.e., active global stretching) of 4 min will be performed at the end of the session including five anterior or posterior chain exercises [29]. Total exercise session duration will therefore be 60 min.

The following three weeks of the prehabilitation phase will have a similar structure and organization with some particularities. The aerobic HIIT session total volume will be 32 min organized as follow: (i) warm-up for 4 min at 50–60% pHR, and (ii) four 4 min intervals at 85–95% of pHR interposed by 3 min of active recovery at 65–75% of pHR. Strength HIIT total volume will be 20 min organized as follow: two circuit trainings set (8 min × 2 = 16 min) of eight exercises (30 s work/30 s rest) at 8–9 RPE, with an active between-set rest of 4 min performed at 50–60% of pHR. The same cool-down protocol of 4 min will be also conducted. Total exercise session duration will be therefore of 56 min.

##### Early-Postoperative Phase

This phase will be initialized ≈48 h after the surgical intervention, depending on the surgeon’s advice. All sessions will be performed in the San Cecilio University Hospital (Granada, Spain) and the Laboratory of Exercise Physiology-Sport and Health University Research Institute (iMUDS, Granada, Spain). The exercise intervention during this three week phase will consist on completing: (i) a given number of steps daily (i.e., 1000 steps the first day and increasing 500 steps each subsequent day), and (ii) a similar (but adapted to the surgical intervention) circuit training of strength exercises three times/week maintaining the same structure as the first week of the prehabilitation phase. No instruction about intensity will be provided to the participants in the first week of the early-postoperative phase, while 6–7 RPE and 7–8 RPE will be fixed as the required intensity for the second and the third weeks of this phase, respectively.

##### Late-Postoperative Phase

This phase will have similar characteristics than the last three weeks of the prehabilitation phase. Importantly, since all patients will reach 11,000 steps at the end of the early-postoperative phase, they will be instructed to maintain this premise in the late-postoperative phase, increasing their daily steps by 5% every two weeks.

**Figure 3:**
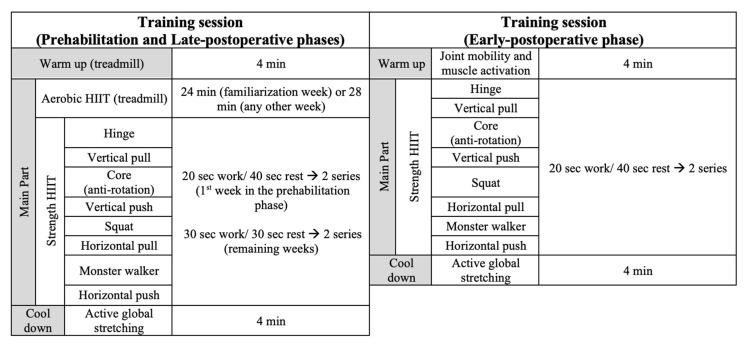
Structure of the implemented concurrent training session within the prehabilitation and postoperative phase. Abbreviations: HIIT; High Intensity Interval Training.

The structure of the implemented concurrent training session within the prehabilitation and postoperative phases can be found in Figure 3.

#### 2.4.2. Dietary Behavior Change

A dietary behavior change intervention is of notorious importance for the treatment of colon cancer, not only for growth prevention but also for avoiding further metastasis [30]. Indeed, malnutrition is considered a relevant risk factor for all-cause mortality in these patients, independently of their age or sex [31], since it has been demonstrated the existence of pro-inflammatory and anti-inflammatory foods able to aggravate or improve, respectively, colon cancer prognostic [32]. Therefore, it seems mandatory to monitor, control, and optimize the dietary patterns before and after colon cancer surgery. Based on previous scientific evidence [33], a dietary behavior change intervention has been designed for the present study including a session per week (i.e., a nutritional talk plus an informative brochure plus a video summary) in the prehabilitation phase (i.e., four weeks) and in the postoperative phase (i.e., 10 weeks).

Nutritional talks will be conducted by a trained nutritionist dietician, their duration being ~15 min in a face-to-face manner, and using audiovisual support. Questions and doubts will be clarified at the end of the session. An informative brochure will be created in order to provide a schematic resume of the main topics covered during the nutritional talks. A video summary will consist of short recording highlighting the key concepts of the nutritional talks. These materials will be completely available for patients and their families, friends, and caregivers.

##### Prehabilitation Phase

Recommendations based on the Mediterranean diet: the 1st (Title: Mediterranean diet) and the 3rd (Title: Dietary fats, sugar and alcohol) sessions will be implemented aiming to offer general and specific recommendations about a healthy and balanced diet based on the Mediterranean diet pattern [34]. Emphasis will be placed on the recommended type of dietary sugar and fat, underling that simple sugar [35] and saturated and trans fats [36] consumption are closely related to a worse progression of colon cancer.

Support for making healthy dietary changes: the 2nd session (Title: Healthy changes) will be performed considering the plate’ method Harvard [37], since previous studies has evidenced the lack of diet variety in the Spanish population diet [38]. Further information about how (i) to organize varied and healthy dishes, and (ii) to discover culinary skills will be offered [39].

Preparation for the surgery: it is well-known that patients who face a surgical intervention with an adequate nutritional status have an enhanced recovery process and longer survival [33]. We will therefore dedicate the 4th session of the prehabilitation phase (Title: Preparation for the surgery) to provide valuable nutritional recommendations for that purpose including: (i) the importance of a proper protein ingestion [40], (ii) the promotion of fluid intake to reach a correct hydration status [41], (iii) the consumption of biocomponents with positive effects on immune system and inflammation process (e.g., omega-3, vitamins or minerals) [42], and (iv) the management of fluid and carbohydrate intake just before the surgery [41].

##### Postoperative Phase

Previous scientific evidence supports the notion that an early nutritional intervention after colon cancer resection is of remarkable importance for ensuring an adequate recovery process and simultaneously decreasing the risk of surgery-derived complications [43]. Similarly, it has been shown that interventions based on nutritional education are an appropriate method not only to optimize the postoperative period but also to improve the long-term quality of life of these patients [44]. Therefore, the dietary behavior change intervention in this phase will consist of:

Nutritional support in the postoperative period and its potential complications: several studies have concluded that patients after colon cancer resection are usually malnourished [45]. Thus, 1st (Title: General postoperative recommendations) and 3rd (Title: Progression of the diet according to tolerance) sessions will focus on giving guidelines for foods selection and the ways of cooking/eating in order to encourage patients to increase the consumption of healthy products and avoid malnutrition [46]. Given that the most common complications in these surgical interventions are diarrhea, constipation, and gases, the 2nd session (Title: Dietary advise for frequent postoperative problems) will be performed aiming to offer nutritional recommendations oriented to these situations [47].

Techniques to improve food hygiene and treatment: malpractice in the treatment and storage of food is one of the leading cause of poisonings in patients who suffer colon cancer [48]. Therefore, the aim of the 4th session (Title: Hygiene and food safety) will be conducted to explain the necessary methods for an appropriate food preservation.

Importance of fiber, microbiota, minerals and vitamins: it is well-demonstrated the key role of microbiota on the recovery process after a colon cancer surgery [49,50]. The 5th session of the postoperative phase (Title: Importance of fiber and microbiota) will be used to explain the importance of microbiota in patient’ health and how fiber ingestion through the diet can help to improve it [51]. Considering that vitamins and minerals are also relevant for maintaining a proper metabolic status [52], the 6th session (Title: Vitamins and Minerals) will summarize nutritional information about which kind of foods contain these elements.

Importance of hydration: Since a correct hydration status is one of the cornerstones of colon cancer patients’ health [52], it has highlighted important problems for the management of minimum water ingestion amounts to maintain a correct hydration status [53]. Thus, the aim of 7th session (Title: Hydration) will be to explain specific strategies to improve fluid intake in our study patients.

Weight management tools through dietary advice: the maintenance of a healthy weight status is recommended after colon cancer resection in order to decrease surgical-derived complications and to increase survival rates [54]. Nutritional advice for weight management will be given to patients in the 8th session (Title: Weight control).

Unhealthy/ultra-processed foods identification and new healthy recipes: numerous studies have reported the deleterious consequences caused by ultra-processed foods and unhealthy dietary habits, with the inflammation process its main driver [55,56]. We will therefore develop the 9th (Title: Ultra-processed foods) and the 10th (Title: Spice up the life) sessions to explain how patients can detect unhealthy and ultra-processed foods, providing alternatives recipes to keep a healthy dietary pattern.

#### 2.4.3. Psychological Support

A comprehensive management of the patient with cancer includes a psychological intervention component as a relevant aspect in disease process and treatment. Medical examinations, waiting for results, the confirmation of the diagnosis and medical treatments entail significant stress situations, not only for patients but also their families. According to previous studies [57,58], up to 40% of patients with cancer develop clinical symptomatology of anxiety or depression during the disease process; this prevalence increasing in situations of disease recurrence. Furthermore, in a 5-year longitudinal study, psychological distress was identified in up to 44% of patients, with higher percentages in men than women [59]. Impaired daily functioning and mood during the disease process and treatment have also been found to adversely impact not only self-management and treatment adherence and functioning, but also symptoms perception and, thus, health-related quality of life [60,61].

Although there is a limited evidence, previous studies in this field of research have shown that a psychological intervention component may be effective at improving psychological distress and the quality of life in adults with colon cancer [62,63,64]. Therefore, a psychological support component will be included in the ONCOFIT intervention. This component will be mainly based on the behavior change model and will include behavior change techniques such as psychoeducation, written and verbal emotional expression, promotion of coping strategies, progressive muscle relaxation training, problem-solving and social skills, and self-efficacy enhancing, among others. This psychological component will also include counseling regarding smoking and alcohol cessation and sleep hygiene.

##### Prehabilitation Phase

We will include a two-session psychological intervention in the month prior to the surgery provided in an individual format and led by clinical psychologists. The objectives of these sessions will comprise the reductions of levels of anxiety, depression, feelings of helplessness and despair, and the increase and promotion of the perception of control, social support, adequate interpersonal communication and, in general, the improvement of the adherence to the other intervention components (medical, nutritional, and physical exercise).

##### Postoperative Phase

The postoperative phase of the psychological support component will include six sessions distributed over the 12 weeks of the intervention in a group format led by clinical psychologists. The main content of this phase is the prevention and/or treatment of potential psychological symptoms related to the disease process and treatment such as anxiety, depression, and psychological distress in general. Therefore, these sessions will include the identification and acceptance of different emotions, and the management of anxiety and depression symptoms after surgery through the use of psychoeducation and the practice of different coping strategies, problem-solving, and social skills. Additional sessions on tobacco and alcohol intake avoidance and cessation will be offered to those patients who are interested. In these sessions, behavior-change techniques such as information on smoking and alcohol intake, self-monitoring, stimulus control, the avoidance of withdrawal symptoms, and relapse prevention, will be used. Sleep hygiene education will also be included at this phase of the psychological support component.

### 2.5. Usual Care/Control Group

All patients recruited for the present randomized controlled trial will follow the usual institutional pre-surgery care as stablished in the Enhanced Recovery After Surgery (ERAS) guidelines [65] including: (i) medication management, (ii) assessment of the surgery-derived risks, and (iii) smoking cessation and peri-operative blood management. Furthermore, a trained nutritionist will evaluate the nutritional status of all patients at the baseline providing oral protein/vitamin supplementation, when necessary, in accordance with the European Society of Parenteral and Enteral Nutrition (ESPEN) guidelines [66]. All consumed oral protein/vitamin supplementation will be appropriately recorded.

Patients allocated to the control group will receive basic expert advice on lifestyle changes from maintaining a proper health status. Concretely, they will be informed about the positive effects of physical exercise, healthy dietary habits, tobacco and alcohol avoidance and psychological health for patients who suffer colon cancer. Furthermore, we will offer the opportunity to receive the ONCOFIT study intervention to patients of the control group after the 1-year follow-up assessment.

### 2.6. Study Endpoints

Socio-demographic and medical variables will be registered at baseline (i.e., five weeks before surgery–week 0) including age, sex, personal and familiar history, surgical history, education levels, social situation, marital status, employment status, level of tumor, cancer stage, and distance of home from hospital.

The primary endpoint of the ONCOFIT study is post-surgery complications determined by the CCI. The main secondary endpoints include: prolonged hospital length of stay, readmissions and emergency department call within 1-year after surgery; functional capacity (assessed by physical fitness); patient reported outcome measures targeted (i.e., health-related quality of life, depression symptoms, anxiety symptoms, mental adjustment to cancer); anthropometry and body composition; clinical/tumor parameters (i.e., blood parameters, clinical characterization, tumor biomarkers, circulating biomarkers, and blood pressure); physical activity levels and sedentariness; dietary habits; others unhealthy habits (i.e., tobacco and alcohol consumptions); sleep quality; and fecal microbiota. The study outcomes will be assessed before randomization at baseline (i.e., five weeks before surgery–week 0), at preoperative conditions (i.e., one day before surgery–week 5), at the end of the PPP intervention (i.e., 12 weeks after surgery–week 17) and 1-year post-surgery (week 57) (Table 2).

#### 2.6.1. Primary Endpoint

The primary outcome of the ONCOFIT study is CCI, an index that accurately and systematically includes all potential complications with their associated grades of severities [67]. It refers to a continuous scale ranging from 0 (i.e., no burden explained by further complications) to 100 (i.e., patient death due to complications) [67]. Post-surgery complications will be assessed at 12 weeks after surgery (i.e., week 17) and 1-year post-surgery (i.e., week 57). They will be graded by severity in accordance with the Clavien-Dindo classification [68]. After grade categorization, post-surgery complications will be converted into the CCI integrating all complications with their associated severities [67]. We will use an online version of the CCI calculator to automatically obtain the patient scores (www.assessurgery.com). It has been previously reported that the CCI is a valid and reliable tool to assess postoperative morbidity and mortality [67]. Indeed, compared with traditional morbidity measures (e.g., overall complications rate or severe complications rate), the CCI constitutes a more comprehensive and sensitive endpoint for surgical investigations [67].

#### 2.6.2. Secondary Endpoints

##### Additional Surgery-Derived Events

Electronic medical records will be used to register data regarding primary and total prolonged hospital length of stay, readmissions, and emergency department appointments within 12 weeks after surgical intervention (week 17) and 1-year post-surgery (i.e., week 57).

##### Functional Capacity

Functional capacity was determined assessing the main components of physical fitness: (i) cardiorespiratory fitness measured by the 6 min walking test (6MWT) distance and gait speed, and (ii) muscular strength assessed by handgrip strength and 5-times sit-to-stand test. The International Fitness Scale will be also used to subjectively assess general physical fitness [69]. These tests will be completed at baseline (week 0), at preoperative conditions (week 5), at the end of the PPP intervention (week 17) and 1-year post-surgery (week 57).

The 6MWT evaluates the ability of an individual to keep a moderate level of walking for a given period of time, therefore reflecting activities of daily living and providing a global evaluation of the physiological demands in response to physical activity at moderate intensity [70]. Patients will be instructed to walk back and forth for six minutes at an intensity that would make them tired by the end of the test, in a 30-m stretch of passage, following the previously published guidelines [71]. A rest stage/period will be allowed during the test, if needed, registering total distance completed at the end of the established time. Previous studies have validated the 6MWT as an accurate indicator of recovery after a colorectal surgical intervention [70,72], considering a change of >20 m as a clinically meaningful improvement [73].

Gait speed has been proved to predict adverse outcomes related to sarcopenia, falls, disability, cognitive impairment and mortality [74,75]. Moreover, it is widely used in practice as it is considered a quick, safe, and highly reliable test for sarcopenia [76,77]. A commonly used gait speed test is called the 4-m usual walking speed being a single cut-off speed ≤0.8 m/s advised by the European Working Group on Sarcopenia in Older People 2 (EWGSOP2) as an indicator of severe sarcopenia [78].

Handgrip strength will be determined using a digital hand dynamometer (TKK 5401 Grip-D; Takei, Tokyo, Japan) and expressed as total kg. Patients will be instructed to perform two attempts for each hand, resting during 1 min between trials. Research staff will encourage them to continuously squeeze for ~3 s exerting their maximal force in all cases. Grip spam of the dynamometer will be fixed at 5.5 cm for men, while a previously validated equation will be implemented for women [79]. The sum of best attempts (left + right hands, respectively) will be considered as the total handgrip strength.

The 5-times sit-to-stand test assess the amount of time needed for a participant to rise five times from a seated position without using arms [80]. This outcome can be used as a proxy for strength of leg muscles (quadriceps muscle group).

The 30-s sit-to-stand muscle power test will record the number of sit-to-stand repetitions performed in 30 s. According to a recently published manuscript, it will provide a valid measure of bilateral lower limb power and a robust assessment of physical performance [81].

##### Patients-Reported Outcome Measures

Colon cancer is expected to impact the daily functioning and mood of patients. The participants’ quality of life will be assessed by a specific questionnaire for patients with colorectal cancer, designed by the European Organization for Research and Treatment of Cancer Quality of Life: the EORTC QLQ-CR29 [82]. We will also assess patients-reported outcome measures by the Beck Depression Inventory-II [83], the State-Trait Anxiety Inventory [84], the Hospital Anxiety and Depression Scale [85], and the Mini--Mental Adjustment to Cancer [86]. These tests will be also completed at baseline (week 0), at preoperative conditions (week 5), at the end of the PPP intervention (week 17) and 1-year post-surgery (week 57).

##### Anthropometry and Body Composition

Anthropometry will be assessed following the standard procedures proposed by the International Society for the Advancement of Kinanthropometry [87]. Weight and height will be measured using a calibrated scale and stadiometer (model 799, Electronic Column Scale, Hamburg, Germany) with patients wearing light clothes. Neck, waist, and hip circumferences will also be assessed. Body composition assessment will be obtained through a full-body dual energy X-ray absorptiometry scanner (Discovery Wi, Hologic, Inc., Bedford, MA, USA) positioning the patient and analyzing results in accordance to the manufacturer’s recommendations. The APEX 4.0.2. software will serve to automatically delineated anatomic regions. Bone mineral density (g/cm^2^), lean mass (kg), fat mass (kg), and visceral adipose tissue (kg), will be body composition endpoints. Anthropometry and body composition evaluations will be completed at baseline (week 0), at preoperative conditions (week 5), at the end of the PPP intervention (week 17) and 1-year post-surgery (week 57).

##### Clinical/Tumor Parameters

Blood Parameters

Blood samples will be drawn from the patient’s antecubital vein in a supine position during the morning after an overnight fasting. Blood parameters will include glycemic profile (i.e., insulin and glucose levels), lipid profile (e.g., triglycerides, total cholesterol, high-density lipoprotein cholesterol (HDL-C), and low-density lipoprotein cholesterol (LDL-C) levels), hepatic transaminases (e.g., alanine transaminase (ALT), and γ-glutamyl transferase (γ-GT) levels), blood red profile and renal profile will be also determined. Spectrophotometry (AU5800, Beckman Coulter, Brea, CA, USA), chemiluminescence immunoassay with paramagnetic particles (UniCel DxI 800, Beckman Coulter, Brea, CA, USA) and ELISA procedures will be used to obtain the above-mentioned parameters.

Clinical Characterization

We will also assess blood pressure in the right arm at rest using an Omrom® HEM 705 CP automatic monitor (OMROM Health-Care Co., Kyoto, Japan), in accordance with the recommendations of the European Heart Society [88].

Since colon cancer has been previously associated with insulin resistance [89], metabolic syndrome [90], non-alcoholic fatty liver disease [91], and cardiovascular disease [92], we will calculate the homeostatic model assessment of insulin resistance index (HOMA) [93], the fatty liver index (FLI) as a validated surrogate marker of non-alcoholic fatty liver disease [94], and a cardiometabolic risk score based on the International Diabetes Federation (IDF) criteria [69].

Tumor Biomarker

Tissue collection: on the day of obtaining the colon tissue sample (i.e., surgical intervention day), it will be examined by anatomo-pathologists, who will provide the fragments for study (i.e., normal, and pathological samples), which will later be deposited in RNA (NanoDrop ND2000 spectrophotometer, Thermo Fisher Scientific, San Jose, CA, USA). Tissue and blood samples will be processed through the Biobank and transferred to the laboratory. A fragment of the tissue samples will be frozen at -80 °C for subsequent isolation of RNA, DNA, and protein (All Prep DNA/RNA/Protein Mini Kit, QIAGEN) and to study the molecular/cellular characteristics. Blood samples will be processed to separate serum, plasma, and peripheral blood mononuclear cells (PBMCs).

PBMCs isolation: plasma will be separated from whole blood by low-speed centrifugation at 1500× *g* for 15 min at 4 °C within 1 h of extraction. PBMCs will be isolated within 2 h after blood draw from 30mL EDTA-treated blood samples. Buffy coats will be diluted 1:2 in PBS, and cells will be separated in 5 mL Ficoll gradient (lymphocyte isolation solution, Rafer) by centrifugation at 2000× *g* for 30 min. PBMCs will be collected and washed twice with cold PBS. Harvested PBMCs will be preserved in liquid nitrogen and stored at −80 °C prior to RNA extraction [95,96,97]. PBMC samples will be collected at baseline (week 0), at preoperative conditions (week 5), at the end of the PPP intervention (week 17) and 1-year post-surgery (week 57)

RNA extraction and quantification: total RNA from tissues and PBMCs will isolated using Direct-zol RNA kit (Zymo Research, Irvine, CA, USA) following manufacturer’s instructions [96,98]. The amount of RNA recovered will be determined and its quality assessed by the NanoDrop ND2000 spectrophotometer (Thermo Fisher Scientific, San Jose, CA, USA). One µg of RNA will be reverse transcribed to cDNA using random hexamer primers with the First Strand Synthesis Kit (Thermo Fisher Scientific, San Jose, CA, USA).

Molecular characterization by qPCR dynamic array based on microfluidic technology: a 96.96 Dynamic Array based on microfluidic technology (Fluidigm, San Francisco, CA, USA) will be used to determine the expression of 96 transcripts in 96 samples, simultaneously, as previously reported [96,98,99,100,101]. Specific primers for human transcripts including cancer biomarkers (e.g., cytokeratins, mucins, villin, B-catenin, etc.), growth factors (e.g., VEGF, etc.), endocrine components (e.g., somatostatin, ghrelin, etc.), inflammatory markers (e.g., IL6, TNFα, etc.), and cellular metabolism elements with potential oncogenic effects (e.g., splicing machinery elements, etc.) will be specifically designed. Primers will be selected using the Primer3 software with selection parameters set to identify primer pairs that: (1) span an intron (when possible), (2) differ by no more that 1 °C in annealing temperature, (3) are at least 20 bp in length, (4) have a GC content between 45 and 55%, but (5) exclude primers that may form primer-dimers. The sequences of selected primers will be used in BLAST (NCBI) searches to check for potential homology to sequences other than the designated target. Initial screening of primer efficiency using real-time detection will be performed by amplifying 2-fold dilutions of RT products, where optimal efficiency will be demonstrated by a difference of one cycle threshold between dilutions and a clear melting peak followed by a graded temperature-dependent dissociation to verify that only one product will be amplified. The thermocycling profile will consist of one cycle of 95 °C for 10 min, 40 cycles of 95 °C for 30 s, 61 °C for 1 min, and 72 °C for 30 s. PCR products will be then column-purified (FAVORGEN Biotech, Vienna, Austria) and sequenced to confirm target specificity. After confirmation of primer efficiency and specificity, the concentration of purified products will be determined, and PCR products were serial diluted to obtain standards containing 1, 10^1^,10^2^, 10^3^, 10^4^, 10^5^, and 10^6^ copies of the synthetic template. Standards will be then amplified by qPCR, and standard curves will be generated using the Stratagene Mx3000p software (Stratagene, La Jolla, CA, USA). The slope of a standard curve for each template examined will be approximately −3.33 (R2≈1), indicating that the efficiency of amplification of our primers is 100%. Preamplification, exonuclease treatment and qPCR dynamic array based on microfluidic technology will be implemented following the manufacturer’s instructions using the Biomark HD System (Fluidigm, CA, USA) and the Real-Time PCR Analysis Software (Fluidigm, CA, USA). Finally, the expression level of each transcript in tissue or PBMC samples will be adjusted by a normalization factor (NF) obtained from the expression levels of three different housekeeping genes using Genorm 3.3 (Wah Lin Nanjing International Company, Nanjing, China). This selection will be based on the stability of these housekeeping genes among the experimental groups to be compared, wherein the expression of these housekeeping genes will not be significantly different among groups.

Circulatory Biomarkers

Inflammatory factors, immunological blood profiles, and hormones, will be also determined using spectrophotometry (AU5800, Beckman Coulter, Brea, CA, USA), chemiluminescence immunoassay with paramagnetic particles (UniCel DxI 800, Beckman Coulter, Brea, CA, USA) and ELISA procedures.

Clinical/tumor parameters will be obtained at baseline (week 0), at preoperative conditions (week 5), at the end of the PPP intervention (week 17) and 1-year post-surgery (week 57).

##### Physical Activity and Sedentariness

The International Physical Activity Questionnaire (IPAQ) has been proposed as a reliable and valid tool to determine physical activity levels and sedentariness in Spanish people [102]. This self-administered questionnaire includes a total of four domains related to physical activity: (i) work-related, (ii) transportation, (iii) housework/gardening, and (iv) leisure-time activity [102,103]. Moreover, the IPAQ comprises sedentary behavior indicators such as time spent sitting [102,103]. Information about domain frequency, time per day, and physical activity intensity, are also considered [102,103]. Physical activity levels and sedentariness will be assessed at baseline (week 0), at preoperative conditions (week 5), at the end of the PPP intervention (week 17) and 1-year post-surgery (week 57).

##### Dietary Habits

Dietary habits will be assessed using a previously validated food frequency questionnaire (FFQ) conducted by a trained nutritionist [104]. Patients will be asked the frequency they have consumed each specific food during the last weeks. Importantly, the nutritionist in charge will emphasize to ensure that information provided by the participant will be oriented to common dietary habits instead of recent dietary changes. The research staff will show and describe to the patients the different portions of food for every FFQ item using photographs (i.e., cups, slices, etc.) in order to enhance the accuracy of the consumed amounts [105]. Subsequently, each FFQ food item will be converted into standardizer portions weight (i.e., consumed amount divided by portion weight).

Based on dietary data from the FFQ, we will calculate the Mediterranean Diet Score in order to determine Mediterranean Diet adherence [106], which is a well-validated and powerful predictor of low risk of suffering chronic pathologies and mortality [106]. The Mediterranean Diet Score is mainly based on eight items (i.e., olive oil, fiber, fruits, vegetables, fish, cereals, meat and alcohol) and ranges from 5-40 [106]. Higher scores indicate higher Mediterranean Diet adherence [106].

Dietary habits will be measured at baseline (week 0), at preoperative conditions (week 5), at the end of the PPP intervention (week 17) and 1-year post-surgery (week 57).

##### Others Unhealthy Habits

A seven day self-reported tobacco and alcohol consumption log will be used to determine smoking and alcohol intake, registering the total number of cigarettes and alcoholic units/day, time, and situation, in which both are consumed, as well as the cigarette type, and alcoholic drink. The Fagerström Test for Nicotine Dependence will be used to measure patients’ nicotine dependence, which is a previously validated scale [107]. Tobacco and alcohol consumption will be evaluated at baseline (week 0), at preoperative conditions (week 5), at the end of the PPP intervention (week 17) and 1-year post-surgery (week 57).

##### Sleep Quality

Sleep quality will be determined by the Pittsburgh Sleep Quality Index (PSQI) scale [108], which is a self-report questionnaire including a total of 19 items grouped in seven component scores (ranging from 0–3): (i) subjective sleep quality (i.e., very good to very bad), (ii) sleep latency (i.e., less than 15 min to more than 60 min), (iii) sleep duration (i.e., more than 7 h to less than 5 h), (iv) sleep efficiency (i.e., more than 85% h sleep/h in bed to less than 65% h sleep/h in bed), (v) sleep disturbances (i.e., no events during the last 4 weeks to more than three events/week), (vi) use of sleeping medications (i.e., no sleep medication to more than three times/week), and (vii) daytime dysfunction (i.e., no problem to important problem). PSQI global score ranges from 0 to 21, with a score higher than five indicating poor sleep quality [108]. PSQI evaluations will be completed at baseline (week 0), at preoperative conditions (week 5), at the end of the PPP intervention (week 17) and 1-year post-surgery (week 57).

##### Fecal Microbiota Analysis

Gut microbiota has emerged as an important environmental factor for some cancers, including colon cancer [49,50]. Actually, the gut microbiota is involved in tumor development, progression, and response to treatment [109,110]. These carcinogenic effects are driven by the production of bacteria metabolites (e.g., bile acids), bacterial toxins (e.g., colibactin), bacterial structural components (lipopolysaccharide), and by the bacterial itself (e.g., pro-carcinogenic bacteria) [110]. Therefore, changes in the abundance of specific bacteria and gut microbiota composition in patients with colon cancer might serve as potential biomarker for screening, prognostication, prediction, and the evaluation of the treatment response [111].

A faecal sample (50–60 g) will be obtained using a sterilized plastic container. Participants will receive a standard faecal sample collection kit together with a step-by-step detailed instruction protocol on how to use it. Faecal samples will be transported from the participants’ house to the research center/hospital in a portable cooler (4 °C) and will be immediately stored at −80 °C until DNA extraction.

Faecal samples will be homogenized using a Stomacher® 400 (A. J. Seward and Co. Ltd., London, UK) and DNA extraction and purification steps will be performed with a commercial kit (QIAamp DNA Stool Mini Kit, QIAGEN, Barcelona, Spain) according to the manufacturer’s instructions. DNA concentration and quality will be assessed with a NanoDrop ND2000 spectrophotometer (Thermo Fisher Scientific, San Jose, CA, USA) and a DS-11 microvolume spectrophotometer (DeNovix Inc, WM, USA), respectively. Subsequently, the purified DNA will by amplified by PCR targeting the V3 and V4 hypervariable regions of the bacterial 16S rRNA gene [112]. The obtained pooled PCR products will be purified using AMPure XP beads (Beckman Coulter, Brea, CA, USA) before its quantification. Finally, the amplicons will be sequenced at MiSeq (Illumina, San Diego, CA, USA) using paired-end (2 × 300 nt) Illumina MiSeq sequencing system (Illumina, San Diego, CA, USA).

Microbial communities will be analyzed from phylum to species, and their relative abundances, expressed as percentages, will be calculated for use its use in subsequent analyses. Regarding diversity indexed, both beta [113] and alpha [113] diversity indexes will be calculated. Alpha indexes will comprises four different proxies: (i) species richness [114], (ii) evenness index [113], (iii) Shannon index [114], and (iv) inverse Simpson index [115]. Finally, the metabolic and functional parameters will the inferred from 16rRNA [116] data through the annotation of microbial genes based on the Kyoto Encyclopedia of Genes and Genomes (KEGG) orthology (KOs).

Faecal samples will be collected at baseline (week 0), at preoperative conditions (week 5), at the end of the PPP intervention (week 17) and 1-year post-surgery (week 57)

### 2.7. Cost-Effectiveness Analysis Outcome

ONCOFIT will include cost-effectiveness analysis (CEA) as a study outcome considering the incremental cost-effectiveness ratio (ICER) of the PPP related to the usual care. We will calculate the ratio of incremental costs and incremental clinical benefits as the additional expenditure required to generate an additional unit of benefit, expressed as cost per quality-adjusted life-year (QALY) added, and calculated as CE = (Cost_2_–Cost_1_)/(QALY_2_–QALY_1_) [117]. EuroQol 5-dimension 5-level (EQ-5D-5L) will be used for QALY estimation [118,119]. With regard to the cost measurements, we will follow the WHO recommendations for estimating costs contemplated in its CEA guidelines such as the cost of providing the intervention and costs of accessing the intervention [120].

### 2.8. Sample Size

In a previous study [121], a 17.3-point mean difference in CCI was found between a group of participants who were physically inactive and a group who were regularly active, both groups undergoing an elective colorectal cancer surgery. Therefore, this study sample size was estimated for an α level of 0.05 and 90% power to detect a 17.3-point difference in CCI (primary outcome) between usual care and PPP groups, considering an σ of 25.2 (the CCI standard deviation of the group of participants who were physically inactive). Based on this data, a sample of 36 participants per group was considered sufficient for our analysis. However, assuming a 20% drop-out rate, we decided to recruit a total sample size of 45 participants for each study group. Thus, a total of 90 participants (*n* ≈ 45 women) will be enrolled in the ONCOFIT study.

### 2.9. Analytical Approach and Statistical Power/Data Management

Descriptive and exploratory preliminary analyses of all the study variables to reveal violations of statistical assumptions, distributions, imbalances between the study groups, associations between study variables, covariates/confounders, amount of missing data, and drop-out patterns, will be performed.

Linear mixed-effects models with individual measures of growth being modeled as the function of randomly assigned group, assessment time, and the interaction between group and time, will be used to assess intervention effects on primary and secondary outcomes [122]. Estimations will be performed using the restricted maximum-likelihood method, including an unstructured covariance matrix to adjust for within-participant clustering resulting from the repeated-measures design. This model will assume that missing values are missing-at-random.

All estimations and analyses will be performed with an intention-to-treat approach (including all participants as originally allocated after randomization) and an additional per-protocol approach. Statistical analyses will be conducted using the statistical program R version 4.0.3 (R Project for Statistical Computing, Vienna, Austria); linear mixed-effects models being performed using the “lme4” package [122].

## 3. Potential Impact of the ONCOFIT Study

Colon cancer is a global health problem affecting more than 1 million patients per year worldwide [1], surgical resection being the elective therapy for its curation [2]. Nevertheless, this surgery procedure usually implies several postoperative complications increasing morbi-mortality risk and hospitalization stays, while decreasing the patient’s quality of life [4,5]. Given those important health-related implications, in addition to colon cancer diagnosis and treatment derived cost, this type of tumor has arisen as a major economic and clinical burden on the health system [123].

A study by Zadlo et al. [124] concluded that, although the economic cost of colon cancer varies depending on several factors (e.g., stage of disease at diagnosis, types of medical services used or patient age, among others), the budget per patient suffering this disease is estimated at USD ~97,000. It has been reported that physical inactivity, dietary unhealthy habits, alcohol or tobacco consumption, and bad sleep patterns, are related not only to a higher incidence of colon cancer, but also to a greater risk of presenting surgical-derived complications [6]. Indeed, Rezende et al. [125] have recently highlighted that the direct costs of colon cancer in Brazil attributable to the lack of physical activity is USD ~23.4 millions/year. Therefore, it seems evident the necessity of lifestyle interventions including physical, dietary and psychological programs to enhance the prognosis of these surgical interventions and to decrease their derived health-related costs [5,12,19].

To the best of our knowledge, the ONCOFIT randomized controlled trial is the first study to describe the influence of a multidisciplinary prehabilitation program combined with a postoperative intervention on primary and secondary post-surgery complications in patients undergoing resection of colon cancer. The present study will also investigate their effects on other important physiological and psychological health-related factors in these patients, such as prolonged hospital length of stay, readmissions and emergency department call within 1-year after surgery, functional capacity, patient reported outcome measures, anthropometry and body composition, clinical/tumor parameters, physical activity levels and sedentariness, dietary habits, tobacco and alcohol consumptions, sleep quality, gut microbiota, and circulatory biomarkers. The assessment of all these secondary endpoints will allow us to discover which could be the key outcomes mediating and/or predicting the risk of postoperative complications in patients with colon cancer. The use of objective and subjective determinations of the above-mentioned variables ensuring validity and reliability of our data, in addition to the inclusion of a supervised physical exercise, dietary behavior change, and psychological support on the design and implementation of the multicomponent intervention, provides the ONCOFIT study with unique and robust characteristics in this field of knowledge.

## 4. Conclusions

In summary, the ONCOFIT study will overcome several limitations detected in previous randomized controlled trials involving patients with colon cancer and, therefore, our future results will have a potential clinical and economic impact not only in the scientific community but also in a clinical setting. Considering the feasibility of the present intervention in a real-life scenario, we strongly believe that it would contribute to the standardization of a cost-effective strategy for preventing and improving health-related consequences in patients undergoing resection of colon cancer.

## Figures and Tables

**Table 1:** Inclusion and exclusion criteria.

Inclusion Criteria	Exclusion Criteria
Patients older than 40 years	Medical contraindication for being engaged in an exercise or dietary program.
Diagnostic of nonmetastatic colon cancer (i.e., including right, transverse, left, sigmoid, subtotal, total, and hemicolectomy)	Additional surgery planned within the 3-month intervention
Not participating in a nutritional/dietary intervention	History of another primary invasive cancer
Being physical inactive (i.e., not to be participating in any physical exercise program in the last 3 months, or performing less than 600 metabolic equivalents (METS)/week of moderate-vigorous physical activity).	Planning to receive adjuvant chemotherapy
To be capable and willing to provide informed consent	To be pregnant
Not to suffer from any specific condition that may impede testing of the study hypothesis or make it unsafe to engage in the multidisciplinary intervention (i.e., determined by the research staff)	To present any of the following cardiac conditions: (i) myocardial infarction or coronary revascularization procedure within prior 3 months, (ii) uncontrolled hypertension (i.e., systolic ≥180 mmHg or diastolic ≥100 mmHg), (iii) uncontrolled arrhythmias (iv) valvular disease clinically significant, (v) decompensated heart failure or (vi) to suffer from known aortic aneurysm

**Table 2:** ONCOFIT study endpoints, measurements, and assessments.

Outcome	Measurement	Assessment
Sociodemographic data and medical history		
	Anamnesis	Week 0
	Physical exploration	Week 0
	Sociodemographic interview	Week 0
Surgery-derived events		
	Post-surgery complications	Week 17 and 57
	Hospital length of stay	Week 17 and 57
	Readmissions	Week 17 and 57
	Emergency department appointments	Week 17 and 57
Functional capacity		
Cardiorespiratory fitness	6 min walking test	Week 0, 5, 17 and 57
Gait speed	4 min usual walking speed test	Week 0, 5, 17 and 57
Muscular strength	Handgrip strength	Week 0, 5, 17 and 57
	5-times sit-to-stand test	Week 0, 5, 17 and 57
	30 s sit-to-stand muscle power	Week 0, 5, 17 and 57
Subjective physical fitness	International fitness scale	Week 0, 5, 17 and 57
Patients-reported outcome measures		
Health-related quality of life	EORTC QLQ-C30	Week 0, 5, 17 and 57
Depression symptoms	Beck Depression Inventory-II	Week 0, 5, 17 and 57
Anxiety symptoms	State-Trait Anxiety Inventory	Week 0, 5, 17 and 57
	Hospital Anxiety and Depression Scale	Week 0, 5, 17 and 57
Mental adjustment to cancer	Mini--Mental Adjustment to Cancer	Week 0, 5, 17 and 57
Anthropometry and body composition		
Anthropometry	Weight and height measurement, and neck, waist, and hip circumferences	Week 0, 5, 17 and 57
Body composition	Dual Energy X-ray Absorptiometry	Week 0, 5, 17 and 57
Clinical/tumor parameters		
Blood parameters	Glycemic profile, lipid profile, hepatic transaminases, blood cell profile, and renal function profile	Week 0, 5, 17 and 57
Clinical characterization	Blood pressure, homeostatic model assessment of insulin resistance index (HOMA), fatty liver index (FLI) and the cardiometabolic risk score	Week 0, 5, 17 and 57
Tumor biomarkers	Genetic and molecular biomarkers	Week 0, 5, 17 and 57
Circulatory biomarkers	Inflammatory factors, immunological blood profiles, and hormones	Week 0, 5, 17 and 57
Physical activity and sedentariness		
Physical activity habits	International Physical Activity Questionnaire	Week 0, 5, 17 and 57
Dietary habits		
	Food frequency questionnaire	Week 0, 5, 17 and 57
Mediterranean Diet Adherence	Mediterranean Diet Score	Week 0, 5, 17 and 57
Others unhealthy habits		
Tobacco dependence	The Fagerstrom Test for Nicotine Dependence	Week 0, 5, 17 and 57
Tobacco consumption	Self-reported tobacco consumption logs	Week 0, 5, 17 and 57
Alcohol consumption	Self-reported alcohol consumption logs	Week 0, 5, 17 and 57
Sleep quality		
	Pittsburgh Sleep Quality Index	Week 0, 5, 17 and 57
Fecal microbiota		Week 0, 5, 17 and 57

Week 0 represents baseline assessment; Week 5 represents preoperative conditions assessment; Week 17 represents the assessment after 12-week postoperative intervention; Week 57 represents 1-year post-surgery assessment.

## Data Availability

Not applicable.
